# IgG from *Dermatophagoides pteronyssinus (Der p)*-atopic individuals modulates non-atopic thymic B cell phenotype (alfa-4/beta-7) and cytokine production (IFN-γ, IL-9, and IL-10) with direct membrane interaction

**DOI:** 10.1038/s41598-024-57950-x

**Published:** 2024-03-27

**Authors:** Daniela Terra de-Apoena Reche, Nicolle Rakanidis Machado, Beatriz Oliveira Fagundes, Isabella Siuffi Bergamasco, Thamires Rodrigues de Sousa, Lais Alves do Nascimento, Fernando Roberto Machado Cunha, Marilia Garcia de-Oliveira, Fábio da-Ressureição Sgnotto, Carolina Nunes França, Jefferson Russo Victor

**Affiliations:** 1 Post Graduation Program in Health Sciences, Santo Amaro University (UNISA), São Paulo, SP 04829-300 Brazil; 2 Laboratory of Medical Investigation LIM-56, Division of Clinical Dermatology, Medical School, University of Sao Paulo, Av. Dr. Enéas Carvalho de Aguiar, 500, 3rd Floor, São Paulo, SP 05403-000 Brazil; 3 Ann Romney Center for Neurologic Diseases, Brigham and Women’s Hospital, Harvard Medical School, Boston, MA 02115 USA

**Keywords:** IgG, *Dermatophagoides pteronyssinus*, B cell, IFN-γ, IL-9, IL-22, Alfa-4, Beta-7, Immunology, Humoral immunity

## Abstract

Studies about thymic B cells are scarce in the literature, but it was suggested that they can exert modulatory and regulatory functions on the immune system. Thymic B cells can play some role in regulating the most frequent allergic background worldwide, the atopy induced by the mite *Dermatophagoides pteronyssinus* (Der p). Here, we aimed to evaluate if the polyclonal IgG repertoire produced by Der p-atopic individuals can influence the homing and cytokine profile of human thymic B derived from non-atopic children aged less than seven days. With this purpose, we produced polyclonal IgG formulations and cultivated human thymocytes in their presence. We also assessed IgG subclasses and the direct interaction of IgG with thymic B cell membranes. Our results could demonstrate that Der p-atopic IgG could not reduce the expression of α4β7 homing molecule as observed in response to the other IgG formulations and could reduce the frequency of IFN-γ- and IL-9-producing thymic B cells compared to the mock condition. Der p-atopic IgG could also induce thymic IL-10-producing B cells compared to control conditions. The IgG derived from Der p-atopic individuals failed to diminish the population of IL-13-producing thymic B cells, unlike the reduction observed with other IgG formulations when compared to the mock condition. All IgG formulations had similar levels of IgG subclasses and directly interacted with thymic B cell membranes. Finally, we performed experiments using peripheral non-atopic B cells where IgG effects were not observed. In conclusion, our observation demonstrates that IgG induced in allergic individuals can modulate non-atopic thymic B cells, potentially generating thymic B cells prone to allergy development, which seems to not occur in mature B cells.

## Introduction

Atopy can be characterized as an immune state in which individuals can develop IgE-mediated immune responses, commonly referred to as allergies, in reaction to otherwise harmless antigens known as allergens ^1^. These reactions can be triggered by various groups of allergens, resulting in a range of clinical symptoms, and they arise due to a complex interplay of immunological, genetic, and environmental factors^2^. House dust mites (HDM) are the predominant source of allergens worldwide^3^ and are responsible for clinical symptoms in approximately 50% of asthma patients worldwide^4^. Among the HDM species, Dermatophagoides pteronyssinus (Der p) accounts for roughly 80% of specific IgE antibody reactivity in atopic individuals^4^. This is why our study specifically focuses on individuals with Der p-related allergies.

A Th2 immune response and the production of specific IgE antibodies drive allergies. However, the regulation of these components is intricate and involves various lymphocyte populations, including B cells. Recent evidence suggests B cells may play regulatory or modulatory roles in developing allergic conditions^5^. While human B cell maturation has long been associated with the bone marrow, it was revealed in the early 2000s that the thymus contributes to the peripheral pool of B cells^6^. Studies have shown that approximately 3 × 10^4^ B cells per day migrate from the thymus, where substantial pro- and pre-B cells can be detected. Assessing thymic B cells' phenotype and cytokine production is crucial to understanding their potential modulatory functions. CLA (cutaneous lymphocyte-associated antigen) is the primary molecule for skin-homing expressed by T cells but is also found in B cells^7^. CLA facilitates the homing of B cells to the skin by interacting with vascular E-selectin expressed in oral mucosa venules and in the skin but not in the intestines^8,9^. It has been demonstrated in humans that the induction of integrin α4β7 expression in B cells may be linked to their migration to the intestinal mucosa^10,11^. Effector B cells can produce various cytokines and play a critical role in regulating immunity^12^. In turn, effector T cells can induce Th1 (IFN-γ) and Th2 (IL-4 and IL-13) responses^13^.

Additionally, human B cells can produce IL-17, potentially influencing the development of asthma^14^, and murine B cells can produce IL-9, playing a crucial role in humoral responses^15^. Recently, the "hooks without bait" immunological hypothesis proposed that polyclonal IgG repertoires from different donor groups may play essential roles in modulating the function of thymic and peripheral T and B cells^16^. In this context, polyclonal IgG from individuals with allergies, atopic dermatitis, HIV infection, and HTLV infection has demonstrated in vitro modulatory effects on various thymic and peripheral lymphocytes, including T cells, B cells, γδT cells, iNKT cells, and innate-like cells (ILC) in both humans and mice^17–25^. These effects have the potential to modulate the pathophysiology of these diseases.

The current study aims to identify human thymic B cells and assess the potential of purified IgG from Der p-atopic individuals in modulating their homing and cytokine production profiles. This pilot investigation also explores the interaction between IgG and B cell membranes and extends to peripheral B cells.

## Methods

### Patient samples

Thymus tissues were collected from 12 patients undergoing corrective cardiac surgery at Hospital do Coração (HCor) in Sao Paulo, Brazil. Inclusion criteria encompassed the absence of immunodeficiency, genetic syndromes, or allergic reactions and age below seven days (mean patient age ± standard error: 3.4 ± 0.54 days).

The excision of donor thymus glands was performed when the surgical team deemed it necessary for cardiac intervention, with subsequent clinical evaluations and monitoring of the donors to assess potential impacts on their health. Notably, no clinical effects attributable to postnatal thymectomy were observed in any donors.

Parental allergic backgrounds were assessed, and only infants with non-atopic mothers were included. Serum samples were obtained from male and female blood donors and categorized into three groups based on their atopic profiles for producing polyclonal IgG formulations. These groups were named non-atopic individuals (n-AT; n = 17), atopic individuals unrelated to Der p (nr-AT; n = 16), and Der p atopic individuals (Der p-AT; n = 18). The inclusion criteria for each group of IgG donors were as follows:

n-AT: Clinically confirmed through medical consultation, without significant IgE-specific titers detected for any tested allergens in an immunoblot assay and without reactivity to any tested allergens in a skin prick test (SPT).

nr-AT: Clinically allergic, confirmed by medical consultation, with IgE-specific titers detected for at least two non-HDM tested allergens and reactivity to at least two non-HDM tested allergens in an SPT.

Der p-AT: Clinically allergic to HDM, as confirmed by medical consultation, with IgE-specific titers detected for the Der p allergen and reactivity to the Der p allergen in an SPT.

Participants with severe eczema, dermographism, or recent use of antihistamines, glucocorticosteroids, or other systemic drugs that could influence the SPT results within the last 15 days were excluded. Each thymus or PBMC sample came from a different donor, and the ethics committees at HCor and the School of Medicine at USP approved the study (CAAE: 63361622.7.0000.0068 and 70823623.0.0000.0068). Written consent was obtained from adult participants (blood sample donors) or parents when the participants were minors (thymus donors).

### Allergen sensitization tests

The SPT was conducted as previously described^26^, using a panel of allergens that included *Dermatophagoides pteronyssinus, Dermatophagoides farinae, Aspergillus fumigatus, Penicillium notatum, Alternaria alternata, Canis familiaris, Felis domesticus,* and *Cladosporium herbarum*. Serum antiallergen IgE levels were determined using a multiplex immunoblot assay (EUROLINE Inhalation 2—EUROIMMUN AG, Lubeck, Germany) with the same allergen panel used in the SPT.

### Polyclonal IgG formulations

IgG was purified from pooled serum samples from n-AT, nr-At, and Der p-AT groups using the Melon Gel IgG Spin Purification Kit protocol (Thermo, USA). The purity of IgG, as confirmed previously by our group^20,22–24,27^, exceeded 95%. Purified polyclonal IgG was sterilized using 0.20-micron filters (Corning, Germany) and stored at − 80 °C. IgG concentrations were determined using the Coomassie Protein Assay Reagent (Pierce, USA), and IgG isotypes (IgG1, IgG2, IgG3, and IgG4) were determined by ELISA (IgG Subclass Human ELISA Kit, ThermoFisher, USA) following the manufacturer's instructions. To assess Der p-specific IgG levels, an Enzyme-Linked Immunosorbent Assay (ELISA) was conducted. Polystyrene plates (BD Biosciences) were coated with 5 µg/mL Der p extract (IPI ASAC) in carbonate buffer. Following an 18-h incubation at 4 °C, plates underwent four washes with PBS-Tween® 20 (0.1%; Promega, Madison, WI, USA). Subsequently, plates were blocked for 1 h at room temperature using PBS-T with 1% bovine serum albumin (PBS-T-BSA 1%). After blocking, plates were rewashed, and serum samples (IgG/Der p 1–1:150; IgG/Der p 2–1:300) were added, followed by a 2-h incubation at room temperature. Following another washing step, biotinylated anti-human IgG was added and incubated for 1 h at room temperature. Subsequently, plates were washed and incubated with Avidin-peroxidase solution (eBioscience) for 30 min at room temperature. After a final washing cycle, plates were developed with 50 µL tetramethylbenzidine reagent (BD Biosciences), and the reaction was halted with 50 µL sulfuric acid (30%). Absorbance was measured at 450 nm using a microplate reader (Molecular Devices, Sunnyvale, CA, USA). Sample concentrations were determined in Arbitrary Units per milliliter (AU/mL) by comparing them to a standard curve generated from a serum with elevated levels of Der p-specific IgG.

All technical steps were carried out under sterile conditions, and endotoxin contamination was absent, as confirmed using the Pierce LAL Chromogenic Endotoxin Quantitation Kit (ThermoFisher, USA). Polyclonal IgG formulations were stained using the Zenon Human IgG Labeling Kit (Invitrogen, USA) for IgG-membrane interaction assays, following the manufacturer's instructions. This labeling method is immunoselective, excluding other proteins, including non-IgG antibodies, resulting in specific IgG staining by blocking the IgG Fc portions. Thymocytes were incubated for 30 min with Zenon-labeled IgG or with Zenon labeling and blocking reagents without IgG or unlabeled IgG as controls. This method was standardized with 100 μg/mL of IgG, the optimal concentration determined by the culture experiment results. The IgG-membrane method was validated by prior incubation with the respective unlabeled purified IgG at the same concentration, confirming complete blockage of staining provided by Zenon-labeled IgG.

### Thymic tissue dissociation and cell isolation

Thymocyte dissociation and cell isolation using enzymatic dissociation were previously outlined by our group^19–22,24,28^. Each thymus was cut into small fragments and exposed to an enzymatic solution composed of RPMI medium, collagenase A, DNase I, and fetal bovine serum (FBS). After enzymatic exposure, the fragments were homogenized and filtered. Cell suspensions were washed, and the pelleted cells were resuspended in cold PBS with EDTA and FBS. Low-density fraction (thymocytes) was collected using Ficoll-Paque, and thymocytes were stored in liquid nitrogen. Thawed thymocytes were used for experiments only if their viability exceeded 75%.

### Cell culture

1 × 106 viable thymocytes were cultured in each well of a 48-well culture plate, along with the previously mentioned purified IgG samples at a concentration of 100ug. Cultures were maintained in RPMI 1640 medium containing 10% FBS at 37 °C in 5% CO2 for 3 days. Brefeldin A was added for the last 24 h only to wells evaluating intracellular cytokine production^22,24,29^. Positive (Phorbol 12-myristate 13-acetate—PMA) and negative (mock condition) controls were used for standardization.

### Flow cytometry analyses in thymocytes and PBMC samples

For viability evaluation, the cells underwent incubation with 0.04 μL of LIVE/DEAD® Fixable Red Dead Cell Stain from ThermoFisher (USA), diluted in 1 mL of 1X PBS. This incubation took place at room temperature for 30 min while ensuring protection from light. Afterward, the cells were gently washed and resuspended in 250 μL of 1X PBS. Subsequently, all extracellular assessments were conducted exclusively on viable cells. For extracellular staining, a specific concentration of antibodies, determined through titration for each antibody used, was added to the samples. These samples were then incubated for 30 min at 4 °C. Following this incubation, the samples were thoroughly washed with PBS, and the supernatant was carefully removed. The cells and antibodies were subsequently fixed using a 1% formaldehyde solution for 10 min. Cells cultured in Brefeldin A's presence were subjected to the same extracellular staining protocol to assess intracellular cytokine production. After fixation, stained monoclonal antibodies were added at specific concentrations, as determined by titration. All the antibodies used for staining, as well as the isotype controls, were provided by BD Biosciences (USA). A total of 500,000 events per sample were acquired in the lymphocyte quadrant, determined by their relative size and granularity, using an LSR II Fortessa flow cytometer from BD Biosciences (USA). Compensation was performed utilizing adsorbed microspheres (CompBeads, BD Biosciences, USA) and the identical antibodies that were employed for both extracellular and intracellular staining. The limit of detection for all evaluated cytokines was standardized at 0.150%.

### Statistical analysis

Statistical analysis was done utilizing GraphPad Prism 5.0, developed by GraphPad Software Inc. in La Jolla, CA. Data obtained from in vitro studies were derived from a total of 6 independent experiments, each consisting of 1 to 2 samples. Significance was determined at the *P* ≤ 0.05 level, employing one-way ANOVA with Tukey's post hoc test for multiple comparisons across all groups.

### Ethics statement

The studies involving humans were approved by the review board of the University of São Paulo, Medical School, São Paulo, Brazil, with reference numbers CAAE: 63,361,622.7.0000.0068 and 70,823,623.0.0000.0068. The studies were conducted under local legislation and institutional requirements. The participant's parents or legal guardians provided written informed consent to participate in this study.

## Results

### Polyclonal IgG from Der p-atopic individuals modulates the expression of α4β7 and production of IFN-γ, IL-9, IL-13, and IL-10

**Figure 1:**
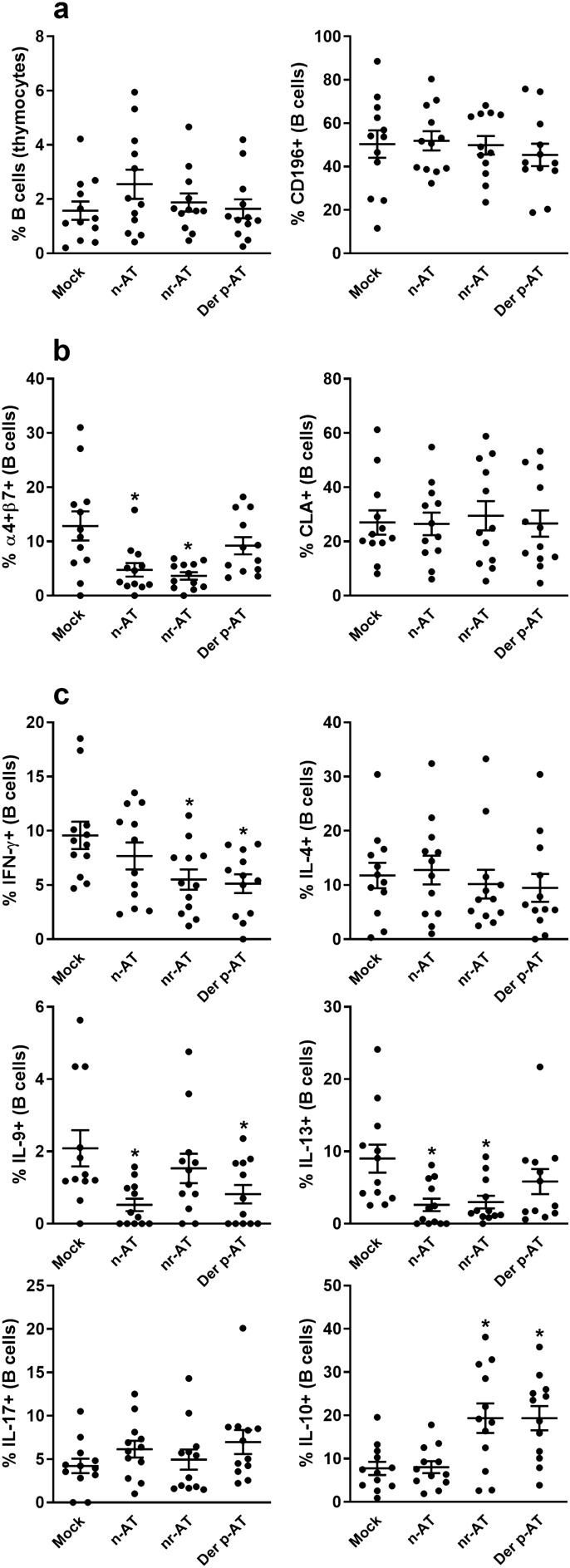
Modulatory effect of IgG from different donors on non-atopic thymic B cells. Thymocytes from non-atopic donors (n = 12) were cultured in the absence of polyclonal IgG (Mock) or the presence of 100 µg/mL of polyclonal IgG from non-atopic individuals (n-AT), allergic patients with atopy to allergens not related to the mite Der p (nr-AT), and allergic patients with atopy to the mite Der p (Der p-AT). After three days of culture, viable B cells were evaluated by flow cytometry for frequency and CD196 expression (**a**), for the expression of the homing molecules α4β7 and CLA (**b**), and for intracellular production of IFN-γ, IL-4, IL-9, IL-13, IL-17, and IL-10 (**c**). Symbols represent individual values obtained from five experiments, and lines represent mean ± SE. * = *p* < 0.05 compared to the Mock condition employing one-way ANOVA with Tukey's post hoc test for multiple comparisons across all groups.

Polyclonal IgG derived from individuals with Der p-atopy was assessed for its influence on α4β7 expression and the production of IFN-γ, IL-9, and IL-13 by thymic B cells. Comparative analyses were conducted with polyclonal IgG from non-related-atopic and non-atopic donors. In vitro experiments involving thymocytes from non-atopic children revealed that the tested conditions did not significantly impact the incidence or expression of the maturation marker CD196 on thymic B cells (Fig. 1a). Moreover, IgG from non-atopic and non-related-atopic donors tended to reduce the frequency of integrin α4β7 on thymic B cells, a phenomenon not observed with Der p-atopic IgG (Fig. 1b). No discernible impact was noted on CLA expression. Concerning cytokine production, IgG from atopic donors (non-related-atopic and Der p-atopic) decreased IFN-γ and increased IL-10 output compared to non-atopic and mock conditions (Fig. 1c). For IL-9 production, both non-atopic and Der p-atopic IgGs diminished cytokine production by thymic non-atopic B cells compared to the mock condition, a response not mirrored by non-related-atopic IgG (Fig. 1c). Furthermore, IgG from non-atopic and non-related-atopic individuals curtailed IL-13 production by thymic B cells, unlike Der p-atopic IgG (Fig. 1c). No effects were observed on IL-4 and IL-17 production across all culture conditions (Fig. 1c).

### Effects of polyclonal IgG on homing and cytokine profiles were unrelated to IgG subclass frequencies but potentially linked to anti-Der p IgG levels and direct IgG membrane interactions

**Figure 2:**
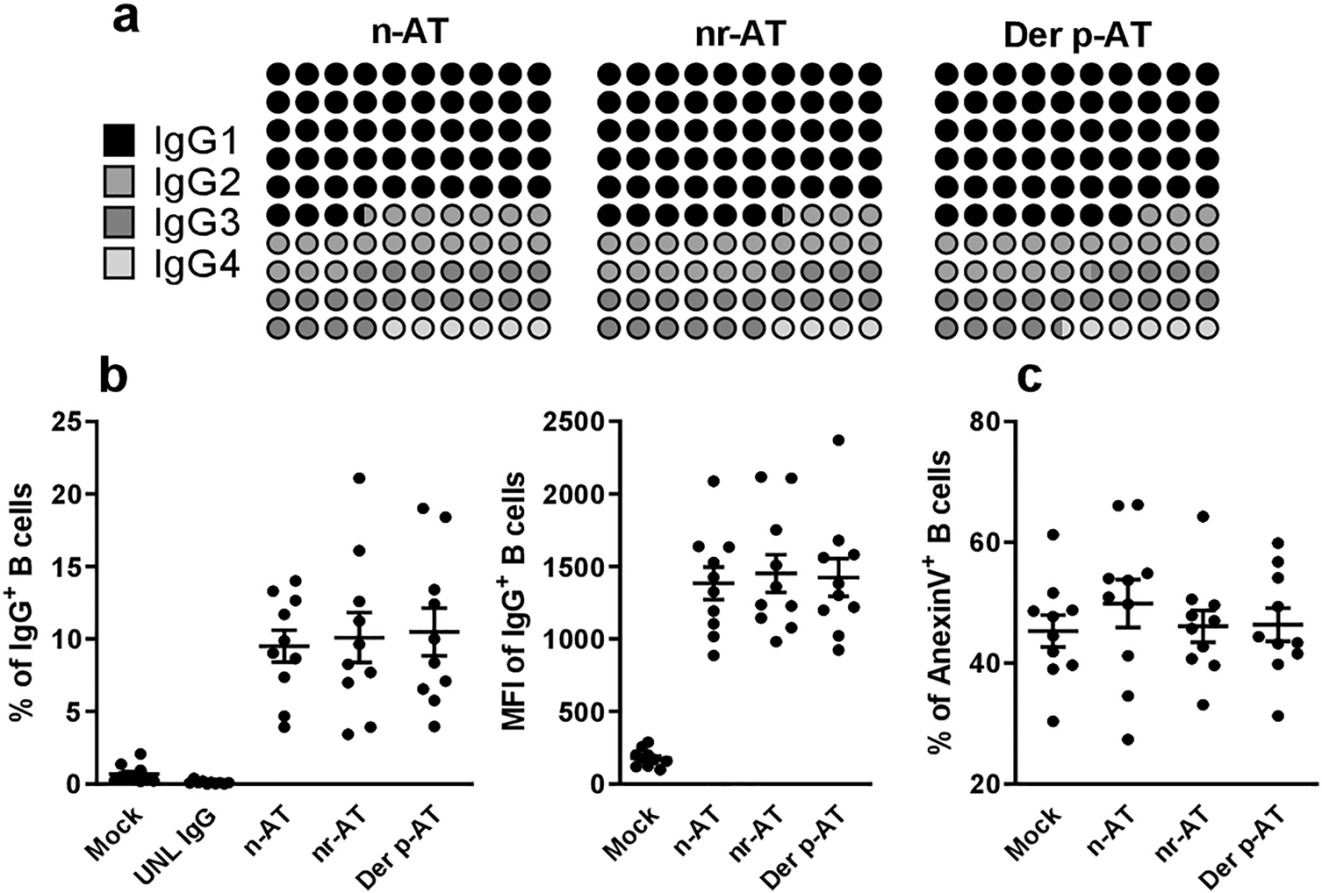
Frequency of IgG subclasses and their direct interaction with thymic B cells. The frequency of IgG1, IgG2, IgG3, and IgG4 isotypes in polyclonal IgG from non-atopic individuals (n-AT), allergic patients with atopy to allergens not related to the mite Der p (nr-AT), and allergic patients with atopy to the mite Der p (Der p-AT) was evaluated (**a**). The frequency and intensity of IgG staining (**b**) and the frequency of Annexin V staining (**c**) was assessed on thymic B cells (n = 10) after incubation with labeling kit reagents (Mock), unlabeled IgG (UNL IgG), labeled n-AT IgG, labeled nr-AT IgG or labeled Der p-AT IgG for 30 min. Symbols represent individual values obtained from five experiments, and lines represent mean ± SE.

Analysis of IgG subclass distributions revealed homogeneity in IgG1, IgG2, IgG3, and IgG4 across all formulations (Fig. 2a). Der p-atopic IgG exhibited significantly elevated levels of anti-Der p IgG (1,458 AU/mL) compared to non-atopic (435 AU/mL) and atopic-non-related (673 AU/mL) donors. Direct interactions between polyclonal IgG and thymic B cell membranes were observed in approximately 10% of cells, with a consistent intensity across all formulations (Fig. 2b). Furthermore, the direct interaction did not precipitate apoptosis, as indicated by comparable phosphatidylserine (PS) expression levels in all tested formulations (Fig. 2c).

### The effects of polyclonal IgG from different donors were not evident in peripheral B cells

**Figure 3:**
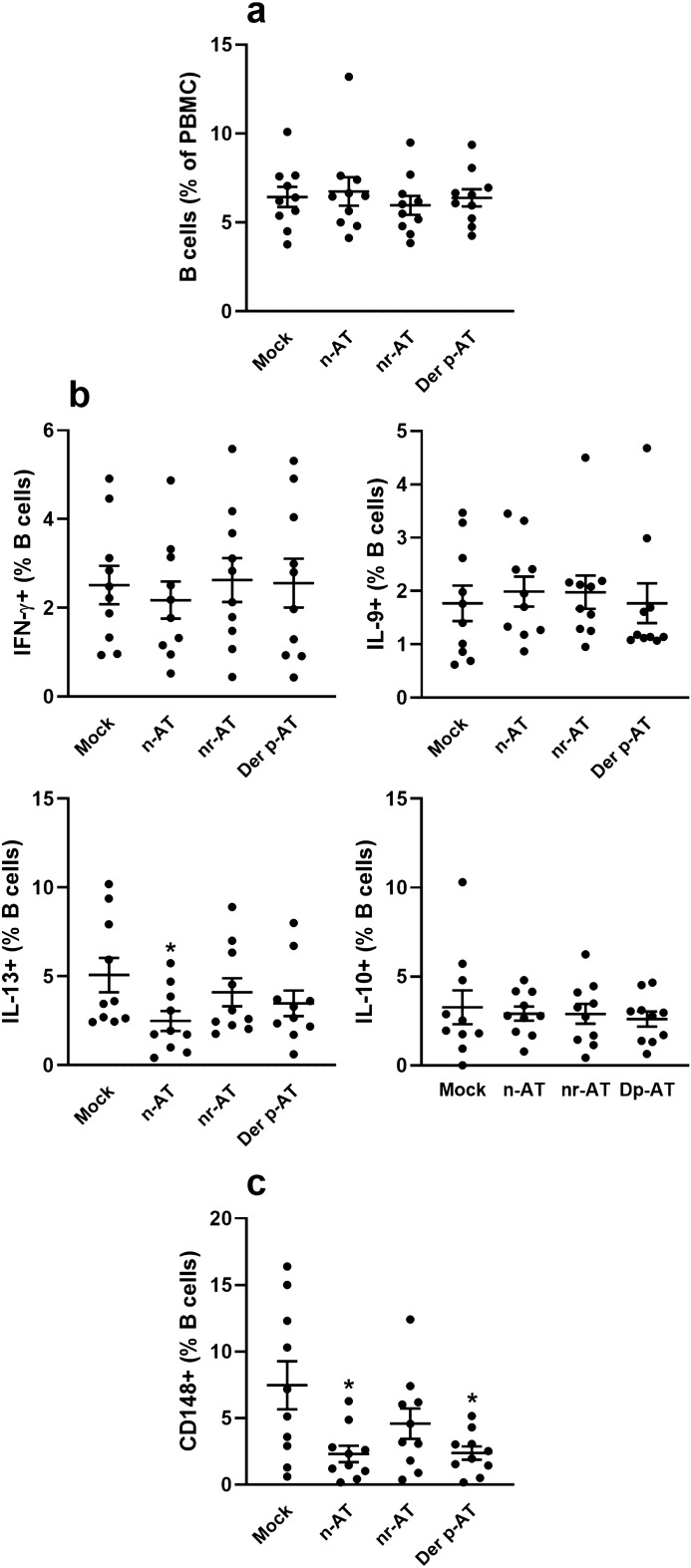
Modulatory effect of IgG from different donors on non-atopic peripheral B cells. PBMCs from non-atopic donors (n = 10) were cultured in the absence of polyclonal IgG (Mock) or the presence of 100 µg/mL of polyclonal IgG from non-atopic individuals (n-AT), allergic patients with atopy to allergens not related to the mite Der p (nr-AT), and allergic patients with atopy to the mite Der p (Der p-AT). After three days of culture, viable B cells were evaluated by flow cytometry for frequency (**a**), for intracellular production of IFN-γ, IL-9, IL-13, and IL-10 (**b**), and the expression of the activation molecule CD148 (**c**). Symbols represent individual values obtained from five experiments, and lines represent mean ± SE. * = *p* < 0.05 compared to the Mock condition employing one-way ANOVA with Tukey's post hoc test for multiple comparisons across all groups.

Substituting non-atopic thymic B cells with non-atopic peripheral B cells in the same culture experiments did not reveal modulatory effects on the intracellular production of IFN-γ, IL-9, IL-13, and IL-10 (Fig. 3a,b). Additionally, the expression of CD148 molecules related to activated and memory B cells demonstrated a lower incidence with polyclonal IgG from non-atopic and Der p-atopic donors compared to mock and atopic-non-related conditions (Fig. 3c).

## Discussion

The study of human thymic B cells presents ongoing challenges due to the relatively recent discovery of their presence in the thymus and practical limitations associated with low B cell frequencies and sample procurement^6^. Previously, our research group demonstrated that the human thymus can generate IL-10-producing regulatory B cells (B10) in response to stimulation with polyclonal IgG from individuals with allergies^18^. In recent years, the immunomodulatory effects of polyclonal IgG have been observed both in vitro^30–34^ and in vivo^35–38^, primarily focusing on T cell populations in murine and human systems.

This study focuses on a critical aspect of human allergen sensitization, the Der p mite, and a relatively unexplored facet of human biology, thymic B cells. We also delve into polyclonal IgG-mediated immunomodulation, a disruptive area in the literature. To achieve this, we generated polyclonal IgG formulations from donors categorized as non-atopic, atopic to non-HDM allergens, or Der p-atopic individuals. We then assessed the impact of these formulations on human thymic B cells. At this juncture, it is pertinent to underscore a methodological aspect. In establishing inclusion criteria, we meticulously assessed the allergic backgrounds of parents, opting to include solely infants with non-atopic mothers. This strategic selection aimed to mitigate potential prenatal exposure of thymus cells to allergen-specific IgG Der p, a factor that could influence the ensuing results.

Our findings indicate that polyclonal IgG derived from Der p-atopic donors did not diminish the expression of integrin α4β7, a molecule associated with cellular homing to mucosal tissues^39^, on thymic B cells, contrary to the effects induced by n-AT and nr-AT IgGs. These results suggest that IgG from Der p-atopic individuals may prevent the reduction of α4β7 expression mediated by other IgG types. Extrapolating these observations to in vivo scenarios, it is conceivable that a repertoire of Der p-atopic IgGs could maintain elevated levels of α4β7 expression on peripheral cells relative to individuals with different IgG backgrounds. Consequently, this phenomenon might facilitate the migration of B cells to mucosal tissues, where clinical manifestations induced by Der p allergens are more likely to occur^40,41^.

We also examined the effect of polyclonal IgGs on key cytokines related to allergic responses. Both atopic-derived polyclonal IgG formulations appeared to reduce IFN-γ production. This reduction may lead to a shift toward a Th2-dominant immune profile, as IFN-γ-producing B cells have the potential to influence the Th1/Th2 balance^13,42^.

Historically, the production of IFN-γ at the airway epithelium has been associated with the regulation of allergic inflammation^43^. However, it has been demonstrated that IFN-γ-producing cells are also present in the lower airways of children with severe allergic asthma, which presents an atypical cytokine profile for an allergic condition^44^. Another important consideration is that in children with severe allergic asthma, eosinophilia and remodeling occur in the absence of Th2 cytokines^45^, suggesting that the reduction of a Th1-related cytokine such as IFN-γ, as observed in our study, may influence secondary mechanisms associated with allergic inflammation. Additionally, it should be noted that severe asthma may be characterized by dysregulated IFN-γ production itself rather than representing a typical indication of a Th1/Th2 balance^46^. This underscores the need for further investigation to precisely elucidate which mechanisms may be influenced by the modulation of IFN-γ, as indicated in our study.

The polyclonal IgG formulations derived from individuals with atopic conditions elicited IL-10 production, suggesting the induction of B10 cells. The induction of thymic B10 cells, facilitated by purified IgG, was initially proposed in the murine thymus. Previous studies have shown that anti-allergen IgG, generated through maternal immunization with OVA, may be associated with the in vivo and in vitro induction of IL-10-producing B cells, specifically B10 cells^23^. Our current study aligns with these findings, demonstrating that thymic B cells from non-atopic donors can be prompted to acquire a B10 phenotype in response to IgG from atopic donors (non-related atopic IgG and Der p-atopic IgG).

Subsequently, a similar protocol, utilizing purified IgG from atopic donors, replicated this effect in human thymocytes some years later^18^. Notably, neither of these studies categorized atopic donors based on their reactivity, a distinction we implemented in our investigation. Considering the pivotal role of thymic B10 cells in maintaining immune homeostasis, exemplified by the substantial suppression of autoimmune responses in lupus-like mice through the up-regulation of Treg cells^47^, our results suggest a potential mechanism for inducing human thymic B10 cells. This implication may hold therapeutic significance, although further elucidation is required.

We observed a distinct modulation of IL-9 production between the two IgG formulations derived from different atopic backgrounds, Der-p-atopic IgG and non-atopic IgG, which demonstrated a reduction in IL-9 production compared to the mock group. This observation suggests a nuanced and unique regulatory influence of various polyclonal IgG formulations on IL-9 production in thymic B cells, an aspect of B cell biology that has received limited attention in the existing literature.

A prior study demonstrated IL-9 production in cord blood B cells and variations between preterm birth (PTB) and term-delivered cases, indicating a potential pro-inflammatory pattern associated with PTB^48^ and unrelated to the atopic background. However, this study did not explore the biological significance of IL-9 secreted by B cells. Another study on human tonsillar B cells did not assess IL-9 production but revealed that IL-9 triggered IL-4-mediated IgE production by B cells by activating cell signaling-related molecules STAT3 and STAT5^49^, suggesting that the IL-9 production by B cells may be related to the atopic background.

Furthermore, murine memory B cells were shown to selectively produce IL-9, exerting autocrine or paracrine effects mediated by IL-9 receptors (IL-9R). These effects are crucial in memory B cell development within germinal centers and the humoral recall response^15,50^ independently of an atopic background or allergen immunization. However, similar effects have yet to be demonstrated in humans.

The limited evidence on the role of IL-9-producing B cells, their relationship with an atopic background, and their impact on the immune response leaves our findings and the modulatory effects mediated by non-atopic and Der p-atopic IgG with ambiguous implications. Therefore, dedicated future studies are essential to address these aspects comprehensively.

Furthermore, we noted that no effect was observed on IL-4 production, and only Der p-atopic polyclonal IgG failed to reduce IL-13 production, both of which are closely associated with the Th2 immune profile^51,52^. These findings suggest that IgG does not induce mechanisms related to the Th2 axis to influence allergy development.

Several aspects were crucial as we explored the possible mechanisms underlying the interaction between IgG and B cells and the observed effects. We investigated potential differences in the frequency of IgG subclasses, specifically IgG4, strongly linked to sensitization to inhalant allergens like Der p^53^. However, no significant differences were detected.

Additionally, we explored whether polyclonal IgG molecules could directly interact with the membrane of thymic B cells, an unprecedented demonstration in the literature. Remarkably, all formulations interacted with approximately 10% of human thymic B cells. Notably, the IgG molecules used in this assay were stained using highly specific anti-IgG Fc portion Fab fragments conjugated to a fluorogen. This blocked the interaction sites of the stained IgG molecules with IgG receptors (FcγRs)^54,55^, indicating that idiotypic interactions may mediate these interactions between polyclonal IgGs and clonal or conserved receptors expressed in the thymic B cell membrane. This aligns with immunological hypotheses suggesting that idiotypic interactions could be responsible for differential effects induced by polyclonal IgG from individuals with varying immune backgrounds^16^.

We also evaluated whether these observed IgG-membrane interactions induced apoptosis in thymic B cells. Our results indicate that none of the tested polyclonal IgGs influenced the frequency of thymic B cells undergoing apoptosis. This suggests that the interactions detected did not induce selective apoptosis as a mechanism responsible for the observed modulatory effects.

Finally, we conducted experiments utilizing peripheral B cells for comparative analysis. Our findings reveal that, except for diminished IL-13 levels mediated by non-atopic IgG, none of the observed effects could be induced in peripheral B cells. The understanding of the development and function of B cells remains incomplete. However, existing literature suggests that thymic B cells may operate through distinct activation mechanisms and exert differential functions compared to their peripheral counterparts^56^. Our results underscore the necessity for comparative studies to elucidate the functionality of thymic B cells. Furthermore, they suggest that strategies for modulating thymic B cells, as demonstrated in this manuscript, can be triggered by non-canonical mechanisms, even without antigens.

In further exploration of peripheral B cells, we briefly assessed the expression of CD148, a molecule co-expressed with CD27 in memory B cells^57^. Notably, both non-atopic and Der p-atopic IgGs could inhibit the expression of CD148 in peripheral B cells, coincidentally the same IgGs responsible for the inhibition of IL-9 in thymic B cells. The reduction in CD148 expression implies a diminished frequency or function of memory B cells. Although speculative, the limited evidence in the literature evaluating the functions of IL-9 in the biology of murine and human B cells suggests that this cytokine may indeed have some effect on the induction of memory B cells^15,49,50^.

While in its early stages, our observation hints at a potential contribution of specific IgG antibody profiles to the induction of IL-9 and the memory phenotype at various stages of B cell maturation. This aspect warrants further elucidation in future studies.

We would like to acknowledge several limitations of our study. While we observed modulation in certain major cytokines associated with the development and regulation of allergies, such as IFN-γ and IL-13, we did not detect any modulation in the production of IL-4 and IL-17, which are also significant cytokines in allergic responses. Additionally, we did not assess IL-5 production, another crucial cytokine related to allergies. Our findings did not align with the classical regulatory pattern of allergy development, where a reduction in IFN-γ typically coincides with the induction of IL-4, IL-13, and IL-17. However, it is plausible that this profile was not observed due to the unsuitability of the classical regulation of Th1 and Th2 cytokine production for B cells. This is supported by the absence of literature classifying B cells into distinct cytokine production patterns.

Furthermore, we propose the hypothesis that B cells may directly migrate to sites of allergic inflammation to exert functional roles, an immunological aspect that remains poorly understood. Recent discussions have highlighted the emerging recognition of B cells as multifaceted contributors to epithelial immunity^58^, suggesting that our observations may gain significance in future research endeavors.

In conclusion, our study sheds light on the role of polyclonal IgG antibodies in interacting with non-atopic human thymic B cell membranes and modulating cytokine production. These interactions may influence the development of an allergic profile but appear less pronounced in mature B cells. Further investigation is needed to fully elucidate these findings' mechanisms and implications.

## Data Availability

The raw data supporting the conclusions of this article will be made available by the corresponding author without undue reservation.
